# Standardizing Access to Sensitive Data with Machine-Actionable Access Conditions

**DOI:** 10.23889/ijpds.v11i4.3816

**Published:** 2026-07-06

**Authors:** Ahmad Hesam

**Affiliations:** 1 SURF, Amsterdam, Netherlands

This paper examines the challenge of governing access to sensitive research data in a scalable and interoperable way. While Trusted Research Environments (TREs) have improved technical protection of sensitive data, access procedures remain fragmented, manual, and largely based on human-readable policies. Using the Dutch social science data landscape as a case study, we identify key governance challenges including inconsistent access conditions, administrative burden, and limited interoperability across data holders.

To address these issues, we propose a framework for machine-actionable access conditions based on the Open Digital Rights Language (ODRL). The framework combines reusable access condition templates with a Data Access Broker service that can interpret conditions and automate parts of the access workflow across repositories and secure analysis environments. Rather than replacing legal or organisational oversight, the approach aims to operationalise existing governance in a more transparent, auditable, and standardised manner.

Although grounded in the Dutch ODISSEI infrastructure, the proposed model is intended to support broader European efforts around EOSC and the European Health Data Space to enable more scalable and interoperable access to sensitive research data.

